# Chronic Obstructive Pulmonary Disease Molecular Subtyping and Pathway Deviation-Based Candidate Gene Identification

**DOI:** 10.22074/cellj.2018.5412

**Published:** 2018-05-28

**Authors:** Jingming Zhao, Wei Cheng, Xigang He, Yanli Liu, Ji Li, Jiaxing Sun, Jinfeng Li, Fangfang Wang, Yufang Gao

**Affiliations:** 1 Department of Respiratory Medicine, The Affiliated Hospital of Qingdao University, Qingdao, China; 2 Department of Respiratory Medicine, People’s Hospital of RizhaoLanshan, Rizhao, China; 3 Department of Pharmacy, Qilu Hospital of Shandong University (Qingdao), Qingdao, China; 4 Department of President’s Office, The Affiliated Hospital of Qingdao University, Qingdao, China

**Keywords:** Chronic Obstructive Pulmonary Disease, Pathway, Subtype

## Abstract

**Objective:**

The aim of this study was to identify the molecular subtypes of chronic obstructive pulmonary disease (COPD) and to prioritize COPD candidate genes using bioinformatics methods.

**Materials and Methods:**

In this bioinformatics study, the gene expression dataset GSE76705 (including 229 COPD samples) and known COPD-related genes (candidate genes) were downloaded from the Gene Expression Omnibus (GEO) and the Online Mendelian Inheritance in Man (OMIM) databases respectively. Based on the expression values of the candidate genes, COPD samples were divided into molecular subtypes through hierarchical clustering analysis. Candidate genes were accordingly allocated into the defined molecular subtypes and functional enrichment analysis was undertaken. Pathway deviation scores were then analyzed, followed by the analysis of clinical indicators (FEV1, FEV1/FVC, age and gender) of COPD patients in each subtype, and prediction models were constructed. Furthermore, the gene expression dataset GSE71220 was used to bioinformatically validate our results.

**Results:**

A total of 213 COPD-related genes were identified, which divided samples into three subtypes based on the gene expression values. After intersection analysis, 160 common genes including transforming growth factor β1 (TGFB1), epidermal growth factor receptor (*EGFR*) and interleukin 13 (IL13) were obtained. Functional enrichment analysis identified 22 pathways such as ‘hsa04060: cytokine-cytokine receptor interaction pathways, ‘hsa04110: cell cycle’ and ‘hsa05222: small cell lung cancer’. Pathways in subtype 2 had higher deviation scores. Furthermore, three receiver operating characteristic (ROC) curves (accuracies >80%) were constructed. The three subtypes in COPD samples were also identified in the validation dataset GSE71220.

**Conclusion:**

COPD may be further subdivided into several molecular subtypes, which may be useful in improving COPD therapy based on the molecular subtype of a patient.

## Introduction

Chronic obstructive pulmonary disease (COPD) is an irreversible or partially reversible disorder with slow progress (1), characterized by progressive airflow obstruction. Patients suffer from this disease for years and die prematurely from it or its complications (2).

Currently, COPD is the fourth major cause of death worldwide and is projected to rank fifth in 2020 (3). Cigarette smoking is generally thought to be a major risk factor for COPD due to the clear association of smoking and airway obstruction (4). However, smokers show considerable interindividual variation in their risk of developing airflow obstruction (5, 6). Interestingly, COPD is found to be more common among relatives of COPD smoker patients than unrelated smokers. Genetics is thus thought to play a role in COPD development (7-9). Therefore, elucidating the underlying genetic etiology may aid the discovery of novel therapeutic targets for this disease. 

Presently, numerous genes have been implicated in the progression of COPD. For instance, alpha 1-antitrypsin deficiency (AATD) is demonstrated to be a clearly inherited risk factor of COPD. Specifically, smokers with AATD have a particularly high risk of developing COPD (10). Additionally, from a molecular perspective, a serial analysis of gene expression by Ning et al. (11) identified stress response genes such as cytokines and chemokines, and pro-apoptotic and anti-proliferation genes to be differentially expressed in COPD patients. Although many genetic factors have been identified, there is no known method for the effective treatment of COPD patients other than improving the symptoms and delaying disease progression (2).

Recently, personalized therapy has been applied in some diseases by subdividing patients into subtypes based on clinical heterogeneity (12). However, Goh et al. (13) reported that COPD has variable clinical phenotypes and it is thus not straightforward to develop individualized treatment programs for patients with this complex chronic disease. We therefore hypothesized that subdividing COPD patients into subtypes based on the expression of identified genetic factors may shed further light onto COPD risk factors and potentially allow personalized therapy in COPD patients.

## Materials and Methods

### Microarray data 

In this bioinformatics study, the gene expression dataset GSE76705 was downloaded from the Gene Expression Omnibus (GEO) database. Individuals analyzed in GSE76705 were Evaluation of COPD Longitudinally to Identify Predictive Surrogate Endpoints (ECLIPSE) subjects donating whole blood as well as peripheral blood mononuclear cells from COPDGene subjects. Gene expression profiling was undertaken on an Affymetrix Human Genome U133 Plus 2.0 Array (HGU133_ Plus_2, Affymetrix Inc., Santa Clara, California, USA). The dataset contained data of 54676 probes in 229 COPD samples. We first normalized the original data using the robust multiarray average (RMA) method in Affy package, calculated the mean value and standard deviation, and then transformed expression values into standard normal distribution using Z-test. We then converted the probes into gene symbols using the Affy package. For multiple probes that mapped to the same gene symbol, their mean value was used as the gene expression value of that gene. 

### Identification of COPD-related genes

COPD-related genes were downloaded from OMIM (http://omim.org/) (14) all of which have key roles in the pathopoiesis of COPD. The Entrez Gene IDs were collected and were then converted into gene symbol. These genes were considered as candidate genes of COPD.

### Unsupervised hierarchical clustering

Based on the expression values of the candidate genes in the 229 COPD samples of GSE76705, we constructed a similarity matrix using hierarchical clustering and the average clustering algorithm. The clustering result was assessed using the cophenetic correlation coefficient and the molecular subtypes of COPD were divided using the 'cutree' function in the R hclust package.

### Subtype-specific gene allocation

After hierarchical clustering, the samples with similar expression profiles were clustered together with patients in different subtypes displaying specific molecular diversities. Since a number of genes may be differentially expressed in different subtypes, we compared the expression levels of candidate genes in different subtypes and allocated the candidate genes into different subtypes. In specific, first we assumed a total of n subtypes were obtained after hierarchical clustering. Next, to determine whether a gene was differentially expressed in a specific subtype, we calculated the P value of differential expression between this subtype and other n-1 subtype using t test. If P<0.05, this gene would be allocated to the subtype which shows a higher level of differential expression (12). Finally, each subtype had its own specific candidate gene set.

### Identification of specific functional pathway and gene of subtype

To investigate the functions of these subtype-specific gene sets, we carried out KEGG pathway enrichment analysis using DAVID (http://david.abcc.ncifcrf.gov/) (15). The enrichment method was based on a corrected Fisher’s Exact Test and pathways with P<0.05 were considered as significantly enriched pathways.

### Pathway deviation score

Since genes specific to different subtypes had different expression patterns, the pathways enriched by these specific genes may have different functional levels in different subtypes, and may thus be targeted in personalized therapies of COPD. Therefore, we quantitatively scored each pathway based on genes enriched in the pathway using equation 1

A(P)=1NΣi=1N(X-i-Y-i)2

Where A(P) represents the deviation score of pathway P, N represents the number of differential genes in P, Xi indicates the average expression value of gene i in the subtypes, and Yi represents the average expression value of gene i in all samples. The deviation level of pathway P in a given subtype was calculated as the cumulative sum of the Euclidean distances of all genes in pathway P. Finally, by comparing the deviation degree of pathway P among different subtypes, we identified: i. The pathways with differences among different COPD subtypes and ii. The associated regulatory genes involved in these pathways (12).

### Analysis of clinical features in molecularly-defined subtypes

The distribution of clinical indicators of COPD, including age, gender, spirometric lung function (FEV1 and FEV1/FVC) and lung parenchymal destruction was compared in different subtypes. Significant differences of these four clinical indicators among the different subtypes were evaluated using analysis of variance (ANOVA) (16).

### Construction of predictive models

Based on the deviation pathways in different subtypes, predictive models of different subtypes were constructed with a tree-based method by using the support vector machine (SVM) (17) classifier. The parameter settings were linear kernel, punish coefficient of 1 and a gamma value of 0. The true positive and false positive values were calculated using a 5-fold cross-validation method. The receiver operating characteristic (ROC) curve was drawn for each subtype, and its stability and accuracy were evaluated by the area under the curve (AUC).

### Bioinformatic-based validation

To validate the COPD molecular subtypes, we downloaded the gene expression dataset GSE71220 (18) from the GEO database, comprising 560 COPD and 57 control samples. Whole blood gene expression of COPD patients from the ECLIPSE study was analyzed using the Affymetrix Human Gene 1.1 ST microarray chip. After data preprocessing, as mentioned above, we clustered the 617 samples based on the expression of COPD-related genes using unsupervised hierarchical clustering analysis. Following that, we used the trained SVM classifier to predict the subtypes of the 617 samples. 

## Results

### Identification of chronic obstructive pulmonary disease-related genes 

A total of 195 Entrez Gene IDs were collected from OMIM, which were converted into 213 gene symbols. According to the expression values of these 213 genes in 229 COPD patients, we constructed the gene expression matrices. After normalization using the Z-Test, all gene expression values followed the normal distribution. 

### Hierarchical clustering analysis

**Fig.1:**
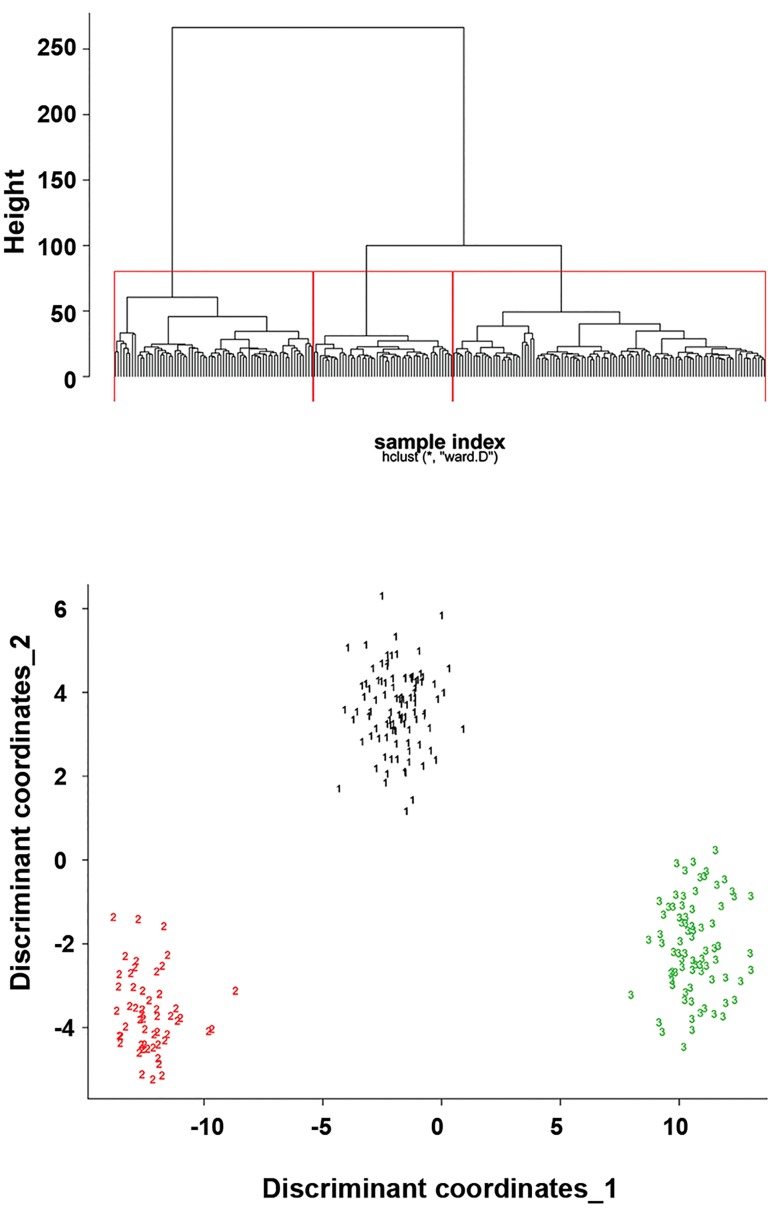
Hierarchical clustering and distribution of chronic obstructive pulmonarydisease (COPD) samples. A. Hierarchical clustering of COPD patients based onCOPD candidate gene expression levels. The horizontal axis represents samplesand the vertical axis (height) represents the distance value between the rightand left sub-branch clusters. The red borders represents the subtypes and B. The sample distribution of the three subtypes. Horizontal and vertical axes areprincipal component coordinates. Black represents subtype 1, red representssubtype 2 and green represents subtype 3.

The 229 samples were divided into three molecular subtypes, as shown in Figure 1A. The cophenetic correlation coefficient was 0.87, indicating no obvious outlier samples or redundant data. Subtypes 1, 2 and 3 contained 98, 53 and 78 samples respectively. The distribution of samples in the three subtypes is shown in Figure 1B.

### Subtype-specific gene allocation 

After allocation of samples into the three subtypes, we obtained three specific gene sets for each of the three subtypes. The number of genes in three gene sets were 166, 170 and 172, respectively. There were 160 common genes, such as transforming growth factor ß1 (*TGFB1*), epidermal growth factor receptor (*EGFR*), interleukin 13 (*IL13*), and B-Raf proto-oncogene, serine/threonine kinase, in the intersection of subtypes 1, 2 and 3.

### Subtype-specific functional pathway analysis

To identify the functions enriched by the specific gene sets, we conducted KEGG pathway enrichment analysis for each subtype, and then selected the common pathways of the three subtypes. A total of 22 common pathways such as ‘hsa05214: Glioma, hsa04060: Cytokine-cytokine receptor interaction’, ‘hsa05222: Small cell lung cancer’ and ‘hsa04110: Cell cycle’ were obtained. Pathways unique to each subtype included hsa04062: Chemokine signaling pathway (subtype 1;* CXCR1* and *CXCR2*), hsa04012: ErbB signaling pathway (subtype 2; *EGFR*), and hsa04630: Jak- STAT signaling pathway (subtype 2; *IL13*), etc.

### Pathway deviation scores

**Fig.2:**
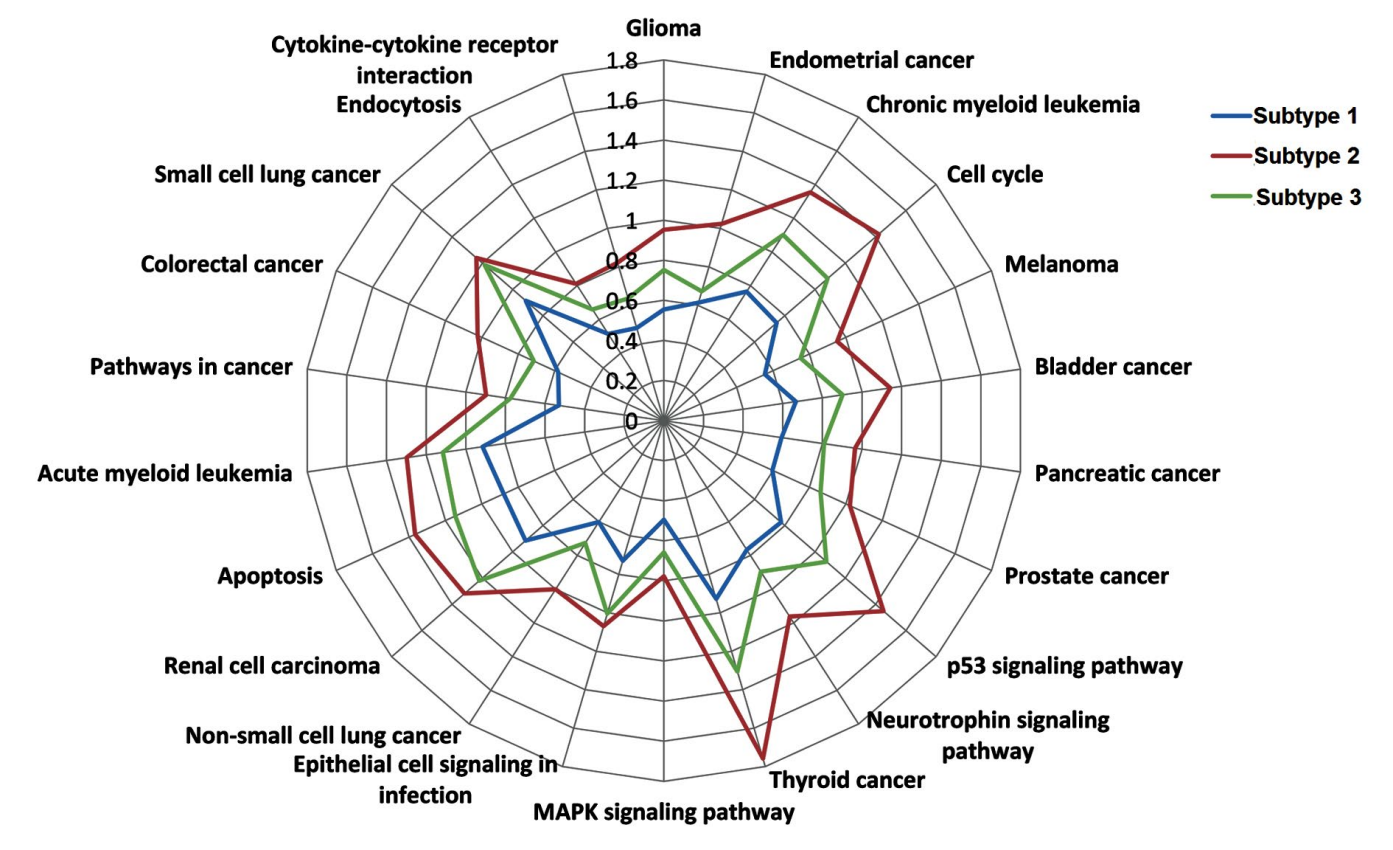
Pathway deviation scores of the subtypes. Subtypes 1, 2 and 3 are marked with blue, red and green lines respectively. Subtype 2 displays the most obvious functional deviation.

To study the functional differences of the common pathways in the three subtypes, we calculated pathway deviation scores in subtypes (Fig .2). The pathways in subtype 2 exhibited the most obvious functional deviation, indicating that patients in subtype 2 may have higher risk for progression compared with subtypes 1 and 3. 

### Subtype-specific clinical feature analysis

**Fig.3:**
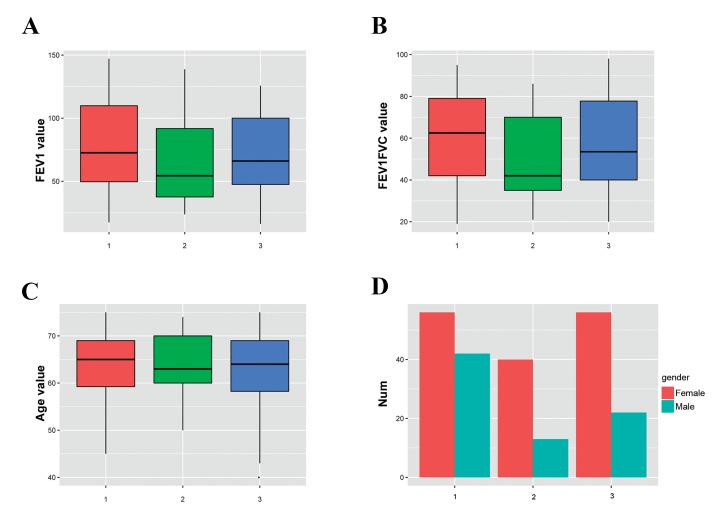
Distribution of four clinical indicators in the three molecular subtypes. A. FEV1 (P=0.03725), B. FEV1/FVC (P=0.01613), C. Age (P=0.073), and D. Gender (P=0.00371).

FEV1 and FEV1/FVC were significantly lower in subtype 2 than that the other two subtypes (Fig .3). P-values of FEV1 and FEV1/FVC differences among the three subtypes were 0.03725 and 0.01613 respectively. There was no significant difference for age in the three subtypes (P=0.073), however, the number of female patients were significantly higher than males in subtypes 2 and 3 (P=0.00371). 

### Predictive model construction 

**Fig.4:**
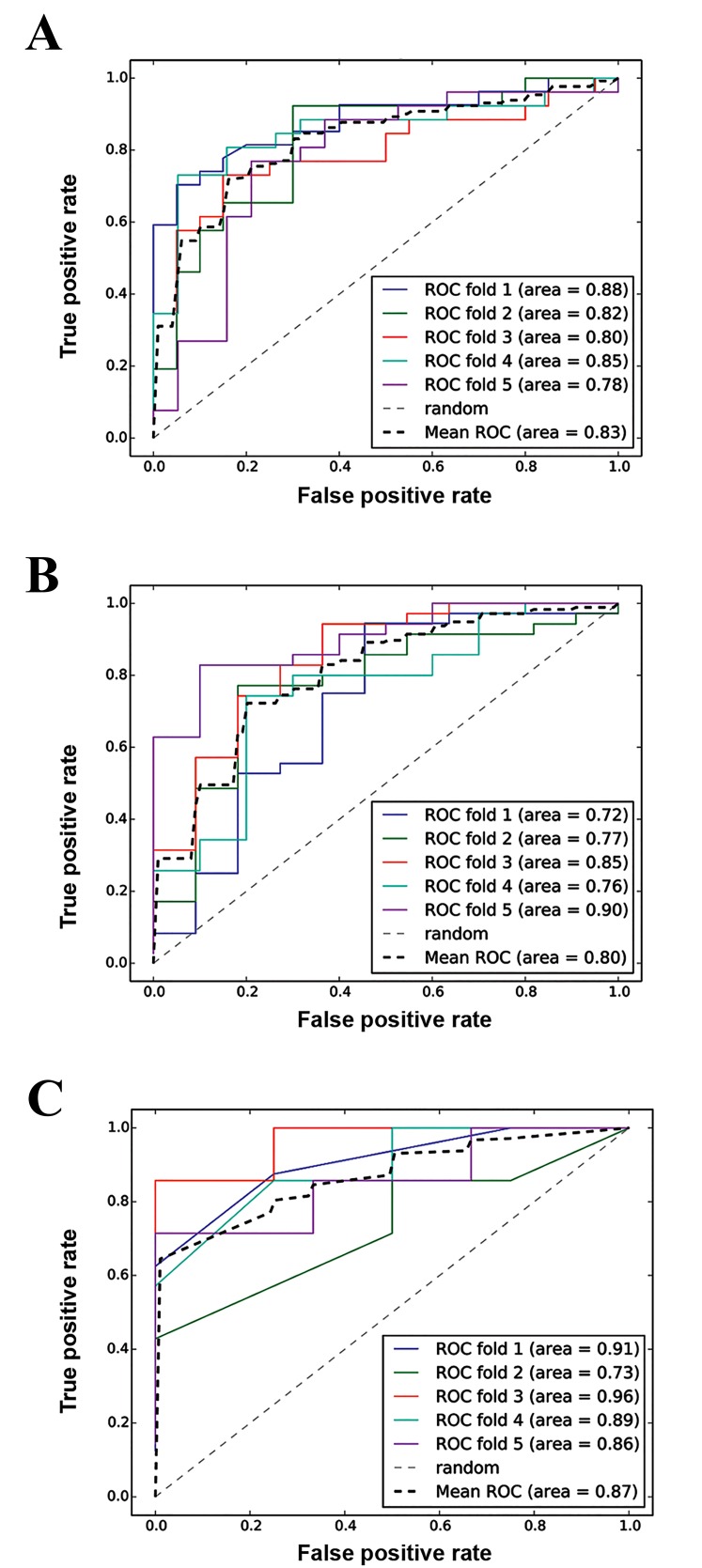
Receiver operating characteristic (ROC) curves of the three subtypes. The average accuracies of the three subtype models (A; 1, B; 2 and C; 3) were 0.83, 0.80 and 0.87 respectively.

On the basis of the 22 common pathways and their deviation scores, we constructed the predictive models using SVM. The ROC curves of the three subtypes (1, 2 and 3) are shown in Figure 4 with their average accuracies being 0.83, 0.80 and 0.87 respectively. 

### Data validation 

**Fig.5:**
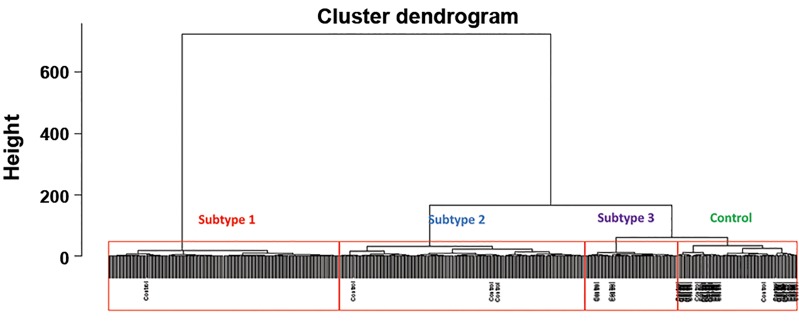
Hierarchical clustering of the GSE71220 dataset. The horizontal axis represents samples and the vertical axis represents height value. Red borders represents the subtypes.

To independently validate the subtypes, we examined the 617 COPD samples from the GSE71220 dataset and identified three subtypes after hierarchical clustering with most of the control samples being clustered in the control group (Fig .5). SVM models were then used to predict the subtypes of COPD patients. As shown in the confusion matrix in Table 1, the consistencies (ratio) of SVM models and hierarchical clustering in the three subtypes and the control group were approximately 70% (63.60%71.70%). To establish that the predicted COPD subtypes were non-random, we calculated the random probability of each subtype achieving the same ratio by randomly sampling samples for 10,000 times. The significant P values of the three subtypes (1, 2 and 3) were 0.0001, 0.0013 and 0.0004 respectively. 

**Table 1:** Comparison of hierarchical clustering and the support vector machine (SVM) model through confusion matrix


Cluster				
SVM model	Subtype 1	Subtype 2	Subtype 3	Control

Subtype1	141	16	11	14
Subtype 2	37	157	16	8
Subtype 3	26	43	56	11
Control	1	3	5	72
Total	205	219	88	105
Ratio	68.70%	71.70%	63.60%	68.60%


## Discussion

These results suggest that although it is difficult for COPD to be clinically subtyped, it can be further divided into subtypes at the molecular level based on candidate gene expression levels, with our predictive models being able to distinguish different subtypes of COPD patients. To the best of our knowledge, this is the first study to subdivide COPD into molecular subtypes. 

COPD is one of the most common inflammatory respiratory diseases (19). A study has reported that cytokines play critical roles in orchestrating the chronic inflammation of COPD by recruiting and activating multiple inflammatory cells in the respiratory tract (20). Cytokines are classified into several types including lymphokines, proinflammatory cytokines, growth factors, and chemokines. In the present study, three genes encoding growth factors (*TGFB1, EGFR*) and lymphokines (*IL13*) were differentially expressed in all COPD patients, all of which are suggested to be implicated in COPD pathogeny and present in the CTD database.

Chemokine signaling pathway (hsa04062) was a unique pathway in subtype 1, which was enriched by *CXCR1* and *CXCR2*. Study has reported that in severe COPD and in an exacerbation in mild COPD, there is an increase in the number of neutrophils in the airways. The neutrophils form a major component of the inflammatory infiltrate in exacerbations of COPD (21, 22). Specially, neutrophils are stimulated and activated through binding of many CXC chemokines to their complementary receptors, notably CXCR1 and CXCR2 (23, 24). Importantly, antagonists targeted against CXCR1 and CXCR2 have been developed for the treatment of COPD (25). Taken together with our study, the pathway of Chemokine signaling pathway as well as CXCR1 and CXCR2 may serve as treatment tergets in subtype 1 COPD.

Jak-STAT signaling pathway (hsa04630) was a unique pathway of subtype 2 and was enriched by *IL13*. IL13 is a Th2 cytokine produced by Th1 CD4^+^T, Th2 CD4^+^ T cells, basophils, etc. It is implicated in recruiting inflammatory cells from the blood to the lung (26). It has been found to play a key role in airway inflammation (27). Zheng et al. (28) have suggested that increased expression of IL13 in the adult murine lung leads to emphysema. They have also revealed that pulmonary expression of transgenic IL13 in adult lungs gives rise to a COPD phenotype with inflammation-dependent emphysema. Importantly, van der Pouw Kraan et al. (29) revealed that human *IL-13* gene was located on a chromosomal region associated with airway high reactivity that was a strong risk factor for COPD. Interestingly, a recent study of Grubek-Jaworska et al. (30) found no significant differences in the level of IL13 between the COPD and asthma groups. Moreover, they found that IL-13 was undetectable in the induced sputum of 6 out of 26 cases of COPD. The different results between our studies may be due to the different tissue samples. Therefore, we speculated that Jak- STAT signaling pathway and *IL-13* might be important candidate targets of subtype 2 COPD.

In addition to 'cell cycle' and 'cytokine-cytokine receptor interaction' pathways mentioned above, 'non-small cell lung cancer' and ’small cell lung cancer' were also identified as specific pathways to COPD. Interestingly, ’small cell lung cancer' had a higher pathway deviation score, suggesting a relationship between COPD and lung cancer. Studies have suggested that nonmalignant pulmonary conditions, such as chronic bronchitis, emphysema and COPD may increase the risk of lung cancer (31, 32). The correlation between COPD and lung cancer has also been assessed from a molecular perspective. 

Lim et al. (33), for instance, reported that COPD was significantly correlated with *EGFR* mutations in non-smoker non-small-cell lung cancer patients. Importantly, the present study shows that *EGFR* was differentially expressed in COPD and was also present in the ‘non-small cell lung cancer’ enriched pathway. Taken together, the pathways associated with lung cancer suggest that COPD is a likely factor of lung cancer development.

Among the three COPD subtypes identified here, subtype 2 had higher pathway deviation scores, suggesting that patients in subtype 2 may have higher risk. In addition, analysis of clinical features showed that FEV1 and FEV1/FVC were also significantly lower in subtype 2. FEV1 provides a straightforward and inexpensive global measurement of airflow limitation and lung function, which is the main intermediate endpoint used in research and for the development of new COPD therapies (34). COPD usually starts in adulthood and causes a rapid decline in FEV1 (35). Additionally, initial airway obstruction is defined when the FEV1/FVC ratio is below the lower fifth percentile of a large healthy reference group (36). Therefore, these results were in accordance with pathway deviation scores, indicating the reliability of the molecular subtypes identified.

Moreover, the ROC curves of the three subtypes had highe average accuracies, indicating that the predictive models have sufficient discriminatory power to distinguish different subtypes of patients. Analysis of the gene expression dataset GSE71220, as a validation dataset, showed that, except for the control group, three COPD subtypes were attainable, further suggesting that COPD may be subdivided into several subtypes. The findings in this study, however, need to be validated with further clinical experiments. We are therefore in the process of collecting COPD samples, such as serum and peripheral blood mononuclear cells, to confirm our results. 

## Conclusion

The present study suggested that COPD could be further subdivided into multiple molecular subtypes. This may be useful in improving COPD therapy based on the molecular subtype of a patient. For instance, subtype 2 patients may require additional treatment given their expression profile being more severely affected. In addition, enrichment of lung cancer related pathways is suggestive of COPD being a risk factor of lung cancer development.
